# Supplementary material for: Big Five Personality Model-based study of death coping self-efficacy in clinical nurses: A cross-sectional survey
Source: PLoS One. 2021 May 27;16(5):e0252430. doi: 10.1371/journal.pone.0252430 (PMC8158956; doi:10.1371/journal.pone.0252430)
Supplement: S1 File — (DOCX) [file pone.0252430.s004.docx]

**Occupational health questionnaire**

**Dear friend, the purpose of this survey is to study the impact of personality traits on the** **death coping self-efficacy of employees. The information does not involve personal privacy issues. Please select the best choice from the options in this table according to your work and health status. Your answers will provide useful references for improving your working conditions and promoting physical and mental health in the future. Please answer every question carefully. The protocol of this study was approved by the ethics committee of Run Run Shaw Hospital. If you don't like it, you can refuse to answer. Thank you very much!**

**Demographic variables：**

1. **Gender：**

①Male ② Female

**2. Age**

**3. Length of service (yrs)**

**4. Marital status**

①Single ②Married ③Single divorced ④Widowed

**5. Educational background：**

①Associate(College) ②Bachelors(University) ③Masters

**6. Religious affiliation**：

①Yes ②None

**7. Received death-related education:**

①Yes ②No

**8. Personal bereavement experience**:

①Yes ②No

**9. The attitude of talking about death**:

①Feeling uncomfortable ②Trying to avoid ③Quite open

**1. Chinese Big Five Personality Inventory brief version (CBF-PI-B)**

**Please answer the following questions according to your actual situation. Only draw a square root on the right answer. You have to answer every question.**

Each item is answered on a six-point Likert-type scale, ranging from 1 (“disagree strongly”) to 6 (“agree strongly”).

| **1** | **2** | **3** | **4** | **5** | | | **6** | |  |
| --- | --- | --- | --- | --- | --- | --- | --- | --- | --- |
| Totally Disagree | Mostly Disagree | A little Disagree | A little Agree | Mostly Agree | | | Totally Agree | |  |
| **1.** I always worry that something bad is going to happen | | | | **1** | **2** | **3** | **4** | **5** | **6** |
| **2.** I often feel scared | | | | **1** | **2** | **3** | **4** | **5** | **6** |
| **3.** Sometimes I feel worthless | | | | **1** | **2** | **3** | **4** | **5** | **6** |
| **4.** I rarely feel melancholy or depressed | | | | **1** | **2** | **3** | **4** | **5** | **6** |
| **5.** I will always contact myself if someone makes a careless sentence | | | | **1** | **2** | **3** | **4** | **5** | **6** |
| **6.** I feel like I'm about to collapse when I'm under pressure | | | | **1** | **2** | **3** | **4** | **5** | **6** |
| **7.** I often worry about trifles | | | | **1** | **2** | **3** | **4** | **5** | **6** |
| **8.** I often feel uneasy inside | | | | **1** | **2** | **3** | **4** | **5** | **6** |
| **9.** I often just want to muddle through and get on with my job | | | | **1** | **2** | **3** | **4** | **5** | **6** |
| **10.** I will continue to work hard to achieve it once the goal determines | | | | **1** | **2** | **3** | **4** | **5** | **6** |
| **11.** I often make a decision after careful consideration | | | | **1** | **2** | **3** | **4** | **5** | **6** |
| **12.** Others think I am a prudent person | | | | **1** | **2** | **3** | **4** | **5** | **6** |
| **13.** One of my characteristics is doing things logically and orderly | | | | **1** | **2** | **3** | **4** | **5** | **6** |
| **14.** I like to plan things from the beginning | | | | **1** | **2** | **3** | **4** | **5** | **6** |
| **15.** I am diligent in my work or study | | | | **1** | **2** | **3** | **4** | **5** | **6** |
| **16.** I work or study very hard | | | | 1 | 2 | 3 | 4 | 5 | 6 |
| **17.** Although there are some bad things in human society (such as war, evil and fraud), I still believe that human nature is generally good | | | | 1 | 2 | 3 | 4 | 5 | 6 |
| **18.** I think most people are well-intentioned | | | | 1 | 2 | 3 | 4 | 5 | 6 |
| **19.** Although there are some frauds in the society, I think most people can be trusted | | | | 1 | 2 | 3 | 4 | 5 | 6 |
| **20.** I don't care much about whether others being treated unfairly | | | | 1 | 2 | 3 | 4 | 5 | 6 |
| **21.** I often feel that the pain of others have nothing to do with me | | | | 1 | 2 | 3 | 4 | 5 | 6 |
| **22.** I often feel sorry for those who encounter misfortune | | | | 1 | 2 | 3 | 4 | 5 | 6 |
| **23.** I am the kind of person who only takes care of myself and does not worry about others | | | | 1 | 2 | 3 | 4 | 5 | 6 |
| **24.** I often feel sad when others tell me misfortune | | | | 1 | 2 | 3 | 4 | 5 | 6 |
| **25.** My imagination is quite rich | | | | 1 | 2 | 3 | 4 | 5 | 6 |
| **26.** My mind is often full of vivid images | | | | 1 | 2 | 3 | 4 | 5 | 6 |
| **27.** I have a strong curiosity about many things | | | | 1 | 2 | 3 | 4 | 5 | 6 |
| **28.** I like adventure | | | | 1 | 2 | 3 | 4 | 5 | 6 |
| **29.** I'm a person who loves to take risks and break the rules | | | | 1 | 2 | 3 | 4 | 5 | 6 |
| **30.** I have a spirit of adventure that no one else has | | | | 1 | 2 | 3 | 4 | 5 | 6 |
| **31.** I am eager to learn some new things, even if they have nothing to do with my daily life | | | | 1 | 2 | 3 | 4 | 5 | 6 |
| **32.** I am willing and easy to accept those new things, new ideas, new ideas | | | | 1 | 2 | 3 | 4 | 5 | 6 |
| **33.** I like to go to social and recreational parties | | | | 1 | 2 | 3 | 4 | 5 | 6 |
| **34.** I'm bored by parties with lots of people | | | | 1 | 2 | 3 | 4 | 5 | 6 |
| **35 .**I try to avoid parties with lots of people and noisy environments | | | | 1 | 2 | 3 | 4 | 5 | 6 |
| **36.** I often act proactively and have fun at parties | | | | 1 | 2 | 3 | 4 | 5 | 6 |
| **37.** I generally not cool when I'm there | | | | 1 | 2 | 3 | 4 | 5 | 6 |
| **38.** I want to be a leader instead of being led | | | | 1 | 2 | 3 | 4 | 5 | 6 |
| **39.** I want to be the leader in a group | | | | 1 | 2 | 3 | 4 | 5 | 6 |
| **40.** Many people think I am a warm and friendly person | | | | 1 | 2 | 3 | 4 | 5 | 6 |

2. **Death Coping Self-Efficacy Scale( DCSS**)

| 1 | 2 | 3 | 4 | 5 | | | | | |
| --- | --- | --- | --- | --- | --- | --- | --- | --- | --- |
| Highly Uncertain | Sometimes Uncertain | Certain | Almost always Certain | Completely Certain | | | | | |
| 1. Being sensitive to the needs of the patient and hid/her family | | | | 0 | 1 | 2 | 3 | 4 | 5 |
| 2. Buy life insurance | | | | 0 | 1 | 2 | 3 | 4 | 5 |
| 3. Listening to the concerns of a dying patient | | | | 0 | 1 | 2 | 3 | 4 | 5 |
| 4. Listening to the family of a dying patient | | | | 0 | 1 | 2 | 3 | 4 | 5 |
| 5. Identify the concerns of a dying patient and his/her family | | | | 0 | 1 | 2 | 3 | 4 | 5 |
| 6. Handle the illness of your child | | | | 0 | 1 | 2 | 3 | 4 | 5 |
| 7. Handle knowing that a family member has a fatal condition | | | | 0 | 1 | 2 | 3 | 4 | 5 |
| 8. Prepare your will | | | | 0 | 1 | 2 | 3 | 4 | 5 |
| 9. Listening to a news report of multiple death | | | | 0 | 1 | 2 | 3 | 4 | 5 |
| 10. Asking to know if someone close to you has a terminal illness | | | | 0 | 1 | 2 | 3 | 4 | 5 |
| 11. Allowing a patient to communicate fully | | | | 0 | 1 | 2 | 3 | 4 | 5 |
| 12. Purchase your cemetery plot | | | | 0 | 1 | 2 | 3 | 4 | 5 |
| 13. Cope with the death of your mother | | | | 0 | 1 | 2 | 3 | 4 | 5 |
| 14. Asking to know if you have a terminal illness | | | | 0 | 1 | 2 | 3 | 4 | 5 |
| 15. Cope with the death of your father | | | | 0 | 1 | 2 | 3 | 4 | 5 |
| 16. Visit a dying friend | | | | 0 | 1 | 2 | 3 | 4 | 5 |
| 17. Providing emotional support for the patient’s family | | | | 0 | 1 | 2 | 3 | 4 | 5 |
| 18. Write a living will | | | | 0 | 1 | 2 | 3 | 4 | 5 |
| 19. Plan your funeral service | | | | 0 | 1 | 2 | 3 | 4 | 5 |
| 20. Attend a funeral or wake where the casket is opened | | | | 0 | 1 | 2 | 3 | 4 | 5 |
| 21. Understand bereavement and grief | | | | 0 | 1 | 2 | 3 | 4 | 5 |
| 22. Cope with the death of your child | | | | 0 | 1 | 2 | 3 | 4 | 5 |
| 23. Handle the death of your spouse | | | | 0 | 1 | 2 | 3 | 4 | 5 |
| 24. Cope with the death of a friend the same age as you | | | | 0 | 1 | 2 | 3 | 4 | 5 |
| 25. Tolerate spiritual and religious differences | | | | 0 | 1 | 2 | 3 | 4 | 5 |
| 26. Cope with the death of a pet | | | | 0 | 1 | 2 | 3 | 4 | 5 |
| 27. Care for myself if I am experiencing stress in caring for a dying patient | | | | 0 | 1 | 2 | 3 | 4 | 5 |
| 28. Being with a person at the time of death | | | | 0 | 1 | 2 | 3 | 4 | 5 |
| 29. Prepay your funeral | | | | 0 | 1 | 2 | 3 | 4 | 5 |

**Each question has five choices. Please tick the number that best reflects your situation.**
