# Supplementary material for: Big Five Personality Model-based study of death coping self-efficacy in clinical nurses: A cross-sectional survey
Source: PLoS One. 2021 May 27;16(5):e0252430. doi: 10.1371/journal.pone.0252430 (PMC8158956; doi:10.1371/journal.pone.0252430)
Supplement: Attachment — Submitted filename: Response to Reviewers.docx [file pone.0252430.s005.docx]

Dear Reviewers,

On behalf of my co-authors, I thank you very much for giving us an opportunity to revise our manuscript entitled “Big Five personality model-based study of death coping self-efficacy in clinical nurses: A cross-sectional survey” (PONE-D-20-32690). We appreciate you very much for your positive and constructive comments and suggestions. Those comments are all valuable and very helpful for revising and improving our paper, as well as the important guiding significance to our researches.

We have carefully considered all issues mentioned in the reviewers' comments, outlined every change made point by point. We have made correction which we hope meet with approval. Revised portion are marked in blue in the paper. The main corrections in the paper and the responds to the reviewer’s comments are as flowing:

Responds to the academic editor comments:

Journal requirements:

1. Whether or not your manuscript can be published will depend, especially, on your response to Reviewer 1's ethical concerns regarding your suggestion that nurses' personality is malleable and should be monitored and shaped by nurse managers. This appears to be a key part of the rationale for your study as well as the most important implication from your results for clinical practice, yet I agree with Reviewer 1 that it is highly problematic from an ethical perspective.

2. Please also take on board comments from both reviewers that highlight the need to edit your manuscript throughout for English.

3. Please ensure that your manuscript meets PLOS ONE's style requirements, including those for file naming.

4. Please clarify in your Methods section whether the questionnaires are published under a CC-BY license, or whether you obtained permission from the publisher to reproduce the questionnaire in this manuscript. Please explain any copyright or restrictions on this questionnaire.

5. We noticed you have some minor occurrence of overlapping text with the following previous publication(s), which needs to be addressed.

6. You may also include additional comments for the author, including concerns about dual publication, research ethics, or publication ethics.

Response:

We appreciate the academic editor very much for his positive comments and suggestions on our manuscript. At the same time, we are very sorry for the expression is not very clear in this paper.

1. We agree with Reviewer 1, and we have deleted “nurses' personality is malleable and should be monitored and shaped by nurse managers” from the article.

2. We have modified grammatical and syntax errors in our article carefully, with the help of an English translator.

3. The manuscript has been modified according to PLOS ONE's style requirements and file naming requirements. We have made correction which we hope meet with approval.

4. Thank you very much for this helpful suggestion. In this study，Chinese Big Five Personality Inventory brief version and Death Coping Self-Efficacy Scale，we wrote to the author of the questionnaire, respectively. Professor Lizhong Zhang and Mengcheng Wang, by E-mail, stating that the use of the scale for academic purpose had been approved by the author. This copyright on this questionnaire has been raised in the Methods section. We have uploaded the CC-BY license in the Attach Files as Supporting Information. The following picture is the screenshot of the email in which Professor Mengcheng Wang and Lizhong Zhang agreed to authorize the use of the questionnaire. Under your suggestion, the email date is January 15, 2020, since the signatures of Professor Wang Mengcheng and Professor Zhang Lizzhong are required and the CC-by license is filled in.

5. We appreciate this suggestion, and we have carefully considered all every change made point by point. We have made correction which we hope meet with approval. Revised portion is marked in blue in the paper.

6. The authors have reviewed the final version of the manuscript and approve it for publication. To the best of our knowledge and belief, this manuscript has not been published in whole or in part nor is it being considered for publication elsewhere.

Responds to the reviewer’s comments:

Reviewer #1:

1. There is no need to include statistics in the abstract. Delete the betas. No need to list tools in the abstract.

2. The authors claim that they examine how personality profiles have an impact on nursing personnel. At the same time they make use of a regression that does not examine impact but only relationship.

3. Please write the word China with a capital letter. For some reason there are sentences where the capital letter appears where it should not. The article requires general editing, it has many small shortcomings of punctuation and inaccuracies. The translation of the article in several places seems to have failed in terms of conveying the original meaning to which the researchers aimed. I recommend using a professional language editor.

4. The authors say: "Moreover, frequent contact between nurses and patients with death-related events can also lead to sympathetic fatigue and exhaustion". There is no such term as sympathetic fatigue. It's compassion fatigue. And this is not exhaustion but burnout. See the article you cited.

5. " The authors say: Nurses who cannot cope with a patient's death may be unable to support dying patients and their families and minimize the quality of hospice care". Why do you think supporting the family is the role of the nurse? Why not of a social worker, a psychologist? Do the nurses in China undergo professional training to provide emotional support?

6. There is no in-depth explanation of personality traits in the article, although this is the main variable

7. Study aims should not appear in the method chapter

8. Study design - not sufficiently described

9. In the article, the researchers deal with associations of demographic and other factors with death coping self-efficacy of nurses, but in practice a large proportion of these variables were not discussed in the literature review.

10. The researchers claim that for nursing students and young nurses, their personality is highly malleable, which is precisely the period to establish a correct outlook on life and values and from independent personality. This is a very strange claim. Do you think it is moral and proper to manage or try to change a person's personality in order to adapt it to the needs of the organization? Do you believe this is possible at all?

11. I feel the researchers included too many variables in the study just because they tested them. It was difficult to find a coherent line of thought in their article. Starting with the presentation of the rationale and its development.

12. Much has been said in the article about what nurses need to do, but there is little reference to the organization's commitment to nurses. For example institutionalized organizational support.

13. It is not clear what all the details in Table 2 are for.

14. Table 4 Factors Affecting death coping self-efficacy (N = 572) - No affect was examined in this study. This is not correct terminology.

15. In summary the researchers claim that they made use of using hierarchical regression. But in Table 4 this is certainly not the way to present a hierarchical regression. Where are the blocks? There is no need to present Tolerance and VI together. This is a multiplicative inverse.

Response #1:

I am very grateful to your comments for the manuscript. They are of great importance to us and our article. According to your advice, we amended the relevant part in manuscript. Some of your questions are answered below.

1. It is really true as the reviewer suggested that “there is no need to include statistics in the abstract. Delete the betas. No need to list tools in the abstract”. Therefore, we deleted the statistics, betas, and tools in Abstract.

2. We appreciate this suggestion, and we have used multiple regression analysis to assess the predictors of personality traits on death coping self-efficacy (Table 5).

3. We have modified grammatical and syntax errors in our article carefully, with the help of an English translator.

4. The former statements are inconsiderate. Under your advice, we rewrote the sentence in the text.

5. Thank you very much for this helpful suggestion. In China, nurses, as the implementer, evaluator and educator of hospice care, play an important role in the comprehensive care of end-stage patients and their families. However, due to resource limitations, there is currently a shortage of psychologists and social care workers or volunteers in multidisciplinary teams in hospitals, and very few nurses are professionally trained to provide emotional support. Different emotional experiences of fearfulness, guilt and self-blame appear to emerge in nurses after patient death in a variety of ways, suggesting that nurses lacked the ability to cope with death. We had also discussed these findings in our paper and showed in the Introduction section.

6. We appreciate the reviewer very much for his positive comments and suggestions on our manuscript. At the same time, we are very sorry for the expression is not very clear in this paper. We have added an explanation of personality in the Introduction and Discussion section.

7. Under your advice, we have deleted the research purpose in the method chapter.

8. Under your advice, we have supplemented study design in the Methods section.

9. We appreciate this suggestion. We have added the relationship between demographic and other factors and death coping self-efficacy of nurses in the Introduction section.

10. We appreciate the reviewer very much for his positive comments and suggestions on our manuscript. At the same time, we are very sorry for the expression is not very clear in this paper. We agree with Reviewer 1, and we have deleted “nurses' personality is malleable and should be monitored and shaped by nurse managers” from the article.

11. Under your advice, we have removed demographic variables from multiple regression analysis, and we have re-written the "introduction" with the rationale and development of death coping self-efficacy and personality traits.

12. This is a very valuable recommendation, we didn’t think about it before. Therefore, we added institutionalized organizational support in the Discussion section.

13. Under your advice, Table 2 has been revised to death coping self-efficacy mean score of nurses.

14. We appreciate this suggestion, and we have modified it to be predictors of nurses’ death coping self-efficacy (Table 5).

15. Thank you very much for this helpful suggestion. We have modified the statistical method to multiple regression analysis (Table 5), and we have deleted VI in Note. Revised portion are marked in blue in the paper.

Reviewer #2:

1. Abstract: in the abstract you mentioned that data were collected in June and July 2020, but in the Methods section, you reported it was done in August and September 2020. So, which is right?

2. The section of introduction is comprehensively organized, but the language needs editing.

3. Methods:

1) Regarding the sample, did you include nurses from pediatric, neonates, and maternity departments? The nurses from these department have different experience and may exhibit a different level of competency on coping with death. You also included ICU nurses, and to my knowledge, these nurses also have different competency of dealing with death. However, I did not understand why you did not collect the data of the departments where nurses work. Why did you include such a different sample but did not analyze the variable of departments, discuss the differences? As you have already finished your data collection, it is impossible to analyze the above questions. Therefore, you may need to mention this as a major limitation in the manuscript.

2) The scale of Death Coping Self-efficacy Scale was translated and revised by a scholar in Taiwan, and it is written in Complex Chinese but not simplified Chinese. More importantly, the people from mainland China where your study was conducted may hold a different perspective in terms of death topics with people in Taiwan. Considering the two facts, I am wondering why you did not translate and test this scale in a sample and report its validity and reliability?

3) Data analysis: in the abstract, you mentioned that you used hierarchical regression analysis, however, in the Methods section, you used multiple regression analysis. From the table, it seemed you used multiple regression analysis. This is contradictory.

4. Results:

What was the mean, SD of score for DCSS? It appears that multiple subgroup analyses based on ANOVA or F tests, t-test or z-test were performed. Given the purpose of the data analyses summarized, I would like to recommend that only multivariate techniques be used such as regression analyses instead of multiple subgroup analyses based on univariate tests in order to assess the contribution of each explanatory variable in estimating the response or outcome variable based on the relationship. Due to the flawed statistical analysis and methods, the article needs to be reworked in a major way and revised before the statistical methods and data analyses can be reviewed and assessed further.

5.Further to the results, the tables were in a chaos. Re-organize them and present them in a clear manner.

Response #2:

We also appreciate the reviewer very much for his positive comments and suggestions on our manuscript.

1. Abstract:

We are very sorry for the expression is not very clear in this paper, and we have amended it in the abstract and the data collection was completed in August and September 2020.

2. We have modified grammatical and syntax errors in our article carefully, with the help of an English translator.

3. Methods:

1）This is a very valuable recommendation, we didn’t think about it before. We have explained a discussion of the limitations of the non-inclusion of pediatric, neonates, and maternity departments.

2) We appreciate the reviewer very much for his positive comments and suggestions on our manuscript. At the same time, we are very sorry for the expression is not very clear in this paper. Before the questionnaire survey, cross-cultural adjustment, pre-survey and validity and reliability test of the scale were carried out, which were not included in the paper before. Sample translations and tests of the scale, and reports on its validity and reliability have been supplemented in the method.

3） Under your advice, we have modified it for multiple regression analysis in the Methods section.

4. Results:

It is really true as reviewer suggested that only multivariate techniques be used such as regression analyses instead of multiple subgroup analyses based on univariate tests in order to assess the contribution of each explanatory variable in estimating the response or outcome variable based on the relationship. Therefore, we have used multiple regression analysis to assess the predictors of personality traits on death coping self-efficacy (Table 5). Demographic variables have been removed from the Table 5 and modified in the corresponding places in the article.

5.We have carefully considered all every change made point by point. We have made corrections which we hope meet with approval. Revised portion are marked in blue in the paper.

We tried our best to improve the manuscript and made some changes in the manuscript. These changes will not influence the content and framework of the paper. And here we did not list the changes but marked in blue in revised paper. We appreciate for Reviewers’ warm work earnestly, and hope that the correction will meet with approval.

Once again, thank you very much for your comments and suggestions.

Looking forward to hearing from you.

Thank you and best regards.

Yours sincerely,

Xi Lin
