# Supplementary material for: Big Five Personality Model-based study of death coping self-efficacy in clinical nurses: A cross-sectional survey
Source: PLoS One. 2021 May 27;16(5):e0252430. doi: 10.1371/journal.pone.0252430 (PMC8158956; doi:10.1371/journal.pone.0252430)
Supplement: Attachment — Submitted filename: Response to Reviewers2.docx [file pone.0252430.s006.docx]

Dear Reviewers,

I am very grateful to your comments for the manuscript. They are of great importance to us and our article. According to your advice, we amended the relevant part in manuscript. Revised portion are marked in red in the paper. The main corrections in the paper and the responds to the reviewer’s comments are as flowing:

Responds to the academic editor comments:

Editor:

1. It is important that in your rebuttal letter, you include the reviewer’s comments as well as your attempt to address them, as in your last letter it was difficult to work out which of the reviewer’s comments each of your responses referred to.

Response: We appreciate the reviewer very much for his positive comments and suggestions on our manuscript. At the same time, we are very sorry for the expression is not very clear in this paper. We have adopted a question-and-answer approach to facilitate the review of editors and reviewers.

2. While I’m pleased to see the authors have changed problematic wording such as “manipulated”, I am still concerned about their rationale that investigating the relationship between personality and self-efficacy in death coping can inform “selecting nurses with appropriate personality traits to join the hospice care team”. This invests far too much confidence in the results of a cross-sectional study that can only provide insight into correlations between a limited range of variable, as well as the validity of the Big 5 Personality Test for measuring personality characteristics most relevant to EOL nursing. After all, the model only explained 20% of the variation in coping, as highlighted in the Discussion. Please remove this idea altogether from both the Introduction, Discussion and Conclusion, and focus on the second part of the rationale regarding education and counselling. The Conclusion in particular will effectively need to be rewritten.

Response: Thank you very much for this helpful suggestion. We have removed the idea in Introduction, Discussion and Conclusion, and have focused on the second part of the rationale regarding education and counselling. ( line 405-425, pages 19 and 20).

The conclusion has been rewritten.

3.There are still instances throughout where the language inappropriately suggests causal relationships that cannot be inferred from cross-sectional data. These include “determined”, “influenced” and “affected”. Strictly speaking, “predict” should also not be used for cross-sectional data since it implies a future time.

Response: Thank you very much for this helpful suggestion, and we have removed the word "predict" from the article.

4. Please use “Big Five Personality Test” or “Model” consistently throughout rather than varying capitalisation and the final word.

Response: Under your advice, we revised the corresponding place in the paper

5. Introduction
Please break up the very long paragraph that begins with “Bandura”.

Response: Under your advice, we broken down a long passage at the beginning of "Bandura.

6.The sentence beginning “In contrast” is difficult to follow.

Response: We have modified the sentence. ( line 102-104, pages 5)

7. “We question whether” is an English phrase usually used to express disbelief, when I think the authors really mean “it is unknown whether”?

Response: Under your advice, we modified it to "it is unknown whether". ( line 144, pages 6)

8. In the sentence beginning “the sense of responsibility” and the following one, please clarify whether these studies were undertaken with nurses.
Response: Under your advice, we clarified that these studies were conducted by nurses. ( line 115-118, pages 5)

9. Discussion
Please rephrase the sentence beginning “For nurses faced with patients” to improve grammar.

Response: Under your advice, we rewrote the sentence beginning “For nurses faced with patients”. ( line 334-335, pages 16)

10.The sentence beginning “The Big Five personality has a significant predictive” needs substantial rewriting to clarify meaning and avoid appearing to be contradictory. Please also remove “poor” as a description of correlation.

Response: Under your advice, we rewrote the sentence that begins with " The Big Five personality has a significant predictive " and changed the word "poor" to "weak." ( line 354-361, pages 17)

11. Please replace “universality” with “generalizability”.

Response: Under your advice, we have replaced "universality" with "generalizability". ( line 429, pages 20)

12. Please add to the limitations section with further acknowledgement that only a limited range of variables were measured, and that some others (as highlighted earlier in the Discussion) might also be important in explaining variability.
Response: Under your advice, we added this note in the limitations section. ( line 435-441, pages 20 and 21)

Responds to the reviewer’s comments:

Reviewer #2:

We also appreciate the reviewer very much for his positive comments and suggestions on our manuscript.

1. On page 2, Introduction, ‘Robbins extracted four dimensions of death coping self-efficacy…’ please provide the reference.

Response: Under your advice, we provided references in the introduction ‘Robbins extracted four dimensions of death coping self-efficacy…’. ( line 72-74, pages 4)

2. In the Introduction, you used ‘hospice care’ and ‘palliative care’. These two are different, and you were supposed to focus on just one term, so you need to delete or change the other. I think you were focusing on palliative care setting. Too much redundant information was provided in Introduction, but little was about personality trait and its relationship with death coping self-efficacy. My suggestion is to shorten this section and focus on what you were doing.

Response: This is a very valuable recommendation, we didn’t think about it before. We removed the 'hospice care', and added information about personality trait and its relationship with death coping self-efficacy. ( line 124-140, pages 6)

3. You were supposed to mark the changes in red, to enable the reviewers easily understand what you revised in the manuscript. I am confused about the documents you provided for further revision, as I am not sure which document is the final one.

Response: We are very sorry for the expression is not very clear in this paper. Revised portion are marked in red in the paper.

4. There is a big flaw---in the Abstract, you mentioned that you focused on palliative care settings, however, in the Methods, you recruited nurses from general wards and ICU. These are not palliative care setting.

Response: We appreciate this suggestion. Because of hospice care in China starts late, only in 2019 opened the first hospice care specialist nurse training, so few hospice care specialist nurse, palliative care environment is not mature, so can only be recruited from the common ward and the ICU nurses, for hospital and community hospice care team, choose a suitable personality traits nurses into the hospice care team, improve the level of hospice care.

5. A competent English editor is recommended.

Response: We have carefully considered all every change made point by point. We have made correction which we hope meet with approval.
