# Supplementary material for: Big Five Personality Model-based study of death coping self-efficacy in clinical nurses: A cross-sectional survey
Source: PLoS One. 2021 May 27;16(5):e0252430. doi: 10.1371/journal.pone.0252430 (PMC8158956; doi:10.1371/journal.pone.0252430)
Supplement: Attachment — Submitted filename: Response to Reviewers3.docx [file pone.0252430.s007.docx]

Dear Reviewers,

I am very grateful to your comments for the manuscript. They are of great importance to us and our article. According to your advice, we amended the relevant part in manuscript. Revised portion are marked in red in the paper. The main corrections in the paper and the responds to the reviewer’s comments are as flowing:

Responds to the academic editor comments:

Editor:

1. Please review your reference list to ensure that it is complete and correct. If you have cited papers that have been retracted, please include the rationale for doing so in the manuscript text, or remove these references and replace them with relevant current references. Any changes to the reference list should be mentioned in the rebuttal letter that accompanies your revised manuscript. If you need to cite a retracted article, indicate the article’s retracted status in the References list and also include a citation and full reference for the retraction notice.

Response: We appreciate the reviewer very much for his positive comments and suggestions on our manuscript. We have reviewed the reference list to ensure that it is complete and correct.

2. Additional Editor Comments (if provided):

I am pleased to see the authors have reduced the inference that personality testing should be used to assess nurses’ suitability for PC. However, there remains an instance of this in the aim (“to provide a basis for selecting nurses with appropriate personality traits to join the palliative care team”) conclusion (“However, palliative care positions should be assigned according to candidates’ personality traits …”) that also need removing. I also suggest removing “to develop appropriate counselling and educational measures to improve the level of palliative care” from the aim and leaving this implication of the research to the Discussion.

Response: Thank you very much for this helpful suggestion. We have removed the inference in the purposes and conclusions that personality testing should be used to assess nurses’ suitability for PC.

We have removed “to develop appropriate counselling and educational measures to improve the level of palliative care” from the aim and leaving this implication of the research to the Discussion. ( line 328-330, pages 18)

3. Similarly, the authors have removed instances of ‘predict’ as requested but not all instances of ‘influence’, most notably from the abstract. This should be replaced by ‘associations between’ to less imply a causal relationship.

Response: Thank you very much for this helpful suggestion, and we have replaced 'influence' with 'associations between'. ( line 28, pages 2)

4. I was interested in the authors’ response to Reviewer #2’s query regarding whether nurses were specialist PC or not. It needs to be made much clearer that there were no SPC services in China until very recently and explained that this is why the study focused on ward/ICU nurses. Some more background and clarification should be inserted around a revised version of the sentence “In China, nurses, as the implementers, evaluators and educators of palliative care, play an important role in the comprehensive care of end-stage patients and their families”.

Then, the aim and Methods need to make clear the focus was on ward/ICU. So, when combined with the suggestions regarding the aim above, the aim should simply read “This study aimed to explore the relationship between personality traits and death coping self-efficacy in nurses from general wards and ICU”.

Response: We are very sorry for the expression is not very clear in this paper. Some more background and clarification have been inserted around a revised version of the sentence “In China, nurses, as the implementers, evaluators and educators of palliative care, play an important role in the comprehensive care of end-stage patients and their families”. ( line 79-90, pages 4 and 5)

This is a very valuable recommendation, we didn’t think about it before.We have made it clear in the aim and methods that the focus is on the ward /ICU. ( line 158, pages 8)

5. Please insert headings into the Abstract – Background, Methods, Results, Conclusions.

Response: Under your advice, we have inserted headings into the Abstract – Background, Methods, Results, Conclusions. ( line 24-43, pages 2)

6. Although the authors have removed some instances of ‘hospice’, there remain some instances in the discussion that need changing to ‘palliative care’.

Response: We appreciate this suggestion. We have changed the word ‘hospice’ in the discussion to ‘palliative care’. ( line 438-447, pages 23)

7. Please remove ‘questionnaire’ from ‘questionnaire survey design’.

Response: Under your advice, we have removed the word ‘questionnaire’ from the ‘questionnaire survey design’.( line 179, pages 9)

8. Please insert an ‘r’ in the word ‘researches’.

Response: Under your advice, we have used the word ‘studies’ instead of ‘researches’. ( line 150, pages 7)

9. I agree with Reviewer 2 that, while the standard of English is generally good, phrasing is frequently a little awkward and would benefit from editing throughout by someone for whom English is a first language.
Response: We apologize for the poor language of our manuscript. We worked on the manuscript for a long time and the repeated addition and removal of sentences and sections obviously led to poor readability. We have now worked on both language and readability and have also involved native English speakers for language corrections. We really hope that the flow and language level have been substantially improved.

10. I would also welcome attempts to shorten the manuscript, which is very long.
Response: We have tried to shorten the text wherever possible, also in line with the editors' comments. Editors are also welcome to shorten manuscripts.
