# Supplementary material for: Big Five Personality Model-based study of death coping self-efficacy in clinical nurses: A cross-sectional survey
Source: PLoS One. 2021 May 27;16(5):e0252430. doi: 10.1371/journal.pone.0252430 (PMC8158956; doi:10.1371/journal.pone.0252430)
Supplement: Attachment — Submitted filename: Response to Reviewers4.docx [file pone.0252430.s008.docx]

Dear Reviewers,

I am very grateful to your comments for the manuscript. They are of great importance to us and our article. According to your advice, we amended the relevant part in manuscript. Revised portion are marked in red in the paper. The main corrections in the paper and the responds to the reviewer’s comments are as flowing:

Responds to the academic editor comments:

Editor:

1. Please remove the following sentence from the Discussion which is misleading about the aim of the study (and which is not normally restated in a Discussion anyway): "The aim was to develop appropriate counseling and educational measures in order to improve the quality of palliative care".

Response: We appreciate the reviewer very much for his positive comments and suggestions on our manuscript. We have removed the following sentence from the Discussion: "The aim was to develop appropriate counseling and educational measures in order to improve the quality of palliative care".
